# Embedding the Mental Capacity Act 2005 in clinical practice: an audit review

**DOI:** 10.1192/pb.bp.114.046870

**Published:** 2014-12

**Authors:** Claudia Dunlop, Oluwatoyin Sorinmade

**Affiliations:** 1 Oxleas NHS Foundation Trust

## Abstract

**Aims and method** An audit cycle assessed compliance of healthcare professionals within Oxleas NHS Foundation Trust with the statutory requirements of the Mental Capacity Act 2005 in patient care. Each stage involved a retrospective review of relevant patient electronic records. The additional purpose of the audit was to make recommendations to improve compliance with the requirement of the Act by healthcare professionals and improve patient understanding of its provisions.

**Results** The audit cycle demonstrated some improvement in clinical practice as well as the need for further efforts at raising the understanding and compliance of clinicians and the public with provisions of the Act.

**Clinical Implications** Healthcare professionals need further understanding of the provisions of the Act and their responsibilities. There is also the need to enhance public awareness to provisions of the Act in relation to their decision-making autonomy. Stakeholders need to put strategies in place for these to be achieved.

The Mental Capacity Act 2005 came into force in 2007 to empower the autonomy of capacitous individuals to make legally binding personal decisions, including decisions to refuse specified treatments in future. It covers diverse aspects of patient care, including issues relating to their property and affairs as well as their personal welfare, and enshrines in law the framework for determining the mental capacity of individuals and the ‘framework for acting and making decisions (best interests decisions) on behalf of individuals who lack the mental capacity to make particular decisions for themselves’.^1^

The Act codifies common law and good practice and legalises instruments such as advance decisions to refuse treatment, lasting power of attorney, court-appointed deputies, and independent mental capacity advocates (IMCAs), among others, to promote the independence of individuals in their decision-making process. The Act creates the Office of the Public Guardian to support the work of the Court of Protection in deciding on all issues relating to mental capacity. It is divided into three parts and is underpinned by five statutory principles which ‘reflect the position that obtained at common law and serve as benchmark for all decisions and actions carried out under the Act’.^2^

## The principles of the Mental Capacity Act

1. The following principles apply for the purposes of this Act.
2. A person must be assumed to have capacity unless it is established that he lacks capacity.
3. A person is not to be treated as unable to make a decision unless all practicable steps to help him to do so have been taken without success.
4. A person is not to be treated as unable to make a decision merely because he makes an unwise decision.
5. An act done or decision made, under this Act for or on behalf of a person who lacks capacity must be done, or made, in his best interests.
6. Before the act is done, or the decision is made, regard must be had to whether the purpose for which it is needed can be as effectively achieved in a way that is less restrictive of the person’s rights and freedom of action.

## Method

In 2009, 2 years after the Mental Capacity Act came into force, an audit was carried out at the Oxleas National Health Service (NHS) Foundation Trust to examine the compliance of clinicians with the prescriptions of the Act during capacity assessment as well as in the process of arriving at the best interests of individuals found to lack the relevant decision-making capacity. A similar process was also carried out at that time at the Princess Royal University Hospital, geriatric department.^3^ The audit standards were derived from the Mental Capacity Act and its Code of Practice.^1^

The initial audit found variable compliance with the Mental Capacity Act requirements and variable understanding of the Act by clinicians and patients alike. The highlights of our findings in terms of areas that required better conformity with requirements of the Act included better documentation and filing of how the decision-making capacity of individuals was determined, better documentation and filing of how the best interests of non-capacitous individuals were determined, and the need for increased awareness of the provisions of the Act by clinicians and the public at large on the essence/role of lasting powers of attorney, court-appointed deputies, advance decision to refuse treatment and involvement of IMCAs where appropriate.

Recommendations were thus made in the following areas:

- that clinicians use a capacity assessment tool to improve the capacity assessment process as well as its documentation; this tool was made available on the Trust’s intranet
- that clinicians use a flowchart also made available on the Trust’s intranet to assist them on the due process in making best interests decisions in the care of non-capacitous individuals
- that clinicians provide patients with information leaflets as a means of enlightening them on the concept of advance decisions as well as the provisions of the Act with regard to ‘capacitous foresight’
- that routine patient clerking should include taking a history from patients on their awareness of provisions of the Act with regard to capacitous foresight.

Three years after the initial audit we again examined the records of a group of patients drawn from within the Oxleas NHS Foundation Trust to determine whether the recommendations of the initial audit had led to better patient care in terms of improved compliance by clinicians with statutory requirements of the Mental Capacity Act. Re-audit data were collected by C.D.

## Results

The re-audit showed improvement in the details of documentation of the capacity assessment process by clinicians in terms of the diagnostic and functional tests involved in this process. Also, the most common reason for which decision-making capacity was assessed in the re-audit was on treatment consenting capacity, whereas in the initial audit the most common reason for capacity assessment was for decisions on place of abode.

There was a marginal increase in the efforts by clinicians to identify whether there was a court-appointed deputy to make decisions on behalf of non-capacitous individuals. In cases where there was a deputy, the clinician followed the decision arrived at after consulting with the deputy. However, there was no improvement in attempts by clinicians to identify whether there was a lasting power of attorney in place. In the cases where there was an effort at identifying the existence of a lasting power of attorney, the re-audit did not show improvement in the number of consultations with the named attorney.

The re-audit showed an increase in the number of cases that were identified as appropriate for the appointment of an IMCA and where an IMCA was identified, the clinicians took into account the report of the IMCA in deciding on the next steps in patient care.

An encouraging finding of the re-audit was that clinical consultation with family members and friends with interests in the patient’s welfare continues to be a central part of the process of arriving at best interest decisions on behalf of non-capacitous individuals. Furthermore, we found an improvement in the details of the entries made by clinicians in patients’ records with regard to the best interest process. Also, detailed entries were made of the best interest process in many more of the cases in the re-audit than at the initial audit.

The re-audit found that there is still a distinct lack of effort in identifying whether an advance decision to refuse treatment was in place. As such, it was not possible to show that any advance decision to refuse treatment (where relevant) was followed or not.

Compared with the initial audit, the re-audit showed a reversal in the ratio of males to females, with more males than females, and there was a slight increase in ‘Other’ ethnic backgrounds. For instance, there were more patients from White Irish and Pakistani ethnic groups than in the initial audit.

## Discussion

The Mental Capacity Act is essentially an empowering piece of legislation, intended to enable individuals to take control of their lives. It enshrines the rights of every citizen to exercise choice and to receive assistance to do so when their ability is limited: quite simply, ‘No decision about me without me’.^4^ The aim is to protect the autonomy of patients to make decisions while also affording protection to adults who might be otherwise vulnerable due to their lack of capacity to make decisions for themselves. The Act explains how to strike a balance between respect for fundamental rights to liberty and autonomy with the need to protect people when they lack capacity to make certain decisions.^5^

We believe that the marginal improvement in some of the practices by clinicians as demonstrated by this audit cycle is partly due to the increasing awareness of the provisions of the Mental Capacity Act within the Trust as well as availability of supportive resources on the Trust’s intranet that facilitate the compliance of clinicians with statutory requirements. The outcome of the re-audit, however, highlights the need for further efforts at raising clinician compliance with prescriptions of the Act. This is in agreement with recent Care Quality Commission reports^5,6^ as well as findings of the House of Lords select committee that scrutinised whether the Mental Capacity Act is working as Parliament intended,^7^ i.e. that a lot of work remains to be done in raising the awareness as well as the understanding of healthcare professionals and the public to the utilisation of this piece of legislation.

We consider that our findings speak to a wider NHS audience given the ongoing initiatives by the Department of Health designed to embed in daily clinical practice statutory requirements contained in the Mental Capacity Act.

The capacity assessment tool designed after the initial audit has since been updated by one of the authors (O.S.) and made available to CSE Healthcare Systems through the London programme for IT Health and Social Care Information Centre (HSCIC) and now features in RiO, the electronic patient care record system used by Oxleas NHS Foundation Trust as well as by some other mental health trusts around the country. O.S. has also worked with HSCIC/CSE Healthcare Systems to update the entire Mental Capacity Act folder on RiO.

### Further recommendations

- There should be regular local workshops on heightening awareness/understanding of staff of the Mental Capacity Act and its ramifications.
- There should be backing by relevant stakeholders for the mandatory use of relevant tools and flowcharts to bolster record-keeping as well as compliance with provisions of the Act.
- Clinicians should provide patients with information leaflets as a means of enlightening them on the concept of advance decisions as well as the provisions of the Act with regard to ‘capacitous foresight’.
- Routine patient clerking should include taking a history from patients on their awareness of provisions of the Act with regard to capacitous foresight.
- There should be regular audits to monitor local compliance with requirements of the Mental Capacity Act.

Our belief is that our recommendations will be relevant to fellow clinicians, the wider community of health professionals and the NHS as a whole and, if implemented, will lead to an improvement in the compliance of clinicians with the requirements of the Mental Capacity Act and maximise the autonomy of patients on decisions relating to their care and treatment.^8^
